# Utility of Cytochemical and Flow Cytometry Detection of Alkaline Phosphatase for Differential Diagnosis of CD34+ Acute Leukaemia in Canines

**DOI:** 10.1111/vco.70024

**Published:** 2025-10-25

**Authors:** Megan Aalto, Janna Yoshimoto, Jillian Nolan, Kenzie Olsen, Dylan Ammons, Emily Rout, Anne C. Avery, R. Adam Harris

**Affiliations:** ^1^ Department of Microbiology, Immunology and Pathology Colorado State University Fort Collins Colorado USA

## Abstract

Alkaline phosphatase (ALP) enzymatic activity has been proposed as a marker for distinguishing canine acute leukaemia (AL) subtypes (i.e., myeloid vs. lymphoid). However, ALP enzymatic activity has not been fully evaluated in CD34+ AL. Determine whether ALP enzymatic activity can differentiate CD34+ AL subtypes in dogs and distinguish CD34+ AL from CD34− haematopoietic tumours in tissue/effusion samples. Peripheral blood from 64 dogs with CD34+ AL, 10 with B cell chronic lymphocytic leukaemia (CLL), and 10 healthy controls were prospectively evaluated for ALP enzymatic activity via cytochemical staining; a subset also underwent ALP detection by flow cytometry (FC). Archived cytology slides from 67 tissue/effusion specimens, including 27 CD34+ AL, 22 T cell lymphomas, and 18 B cell lymphomas, were retrospectively assessed. CD34+ AL cases were categorised as acute myeloid leukaemia (AML), acute lymphoid leukaemia (ALL) or acute unclassifiable leukaemia (AUL) by established FC criteria. ALP positivity was defined as > 3% ALP+ neoplastic cells, which was selected based on receiver operating characteristic (ROC) curve analysis. Cytochemical ALP activity was detected in 61/64 (95.3%) CD34+ AL cases, with no significant differences between AML, ALL, and AUL subtypes (*p* > 0.05). All lymphoma and B cell CLL cases were ALP‐negative. FC‐based ALP analysis showed poor concordance with cytochemistry, and the correlation between %CD34 + ALP+ cells and %ALP+ neoplastic cells was weak (Spearman's *ρ* = 0.25). While ALP enzymatic activity is present in most CD34+ AL cases, it does not reliably differentiate CD34+ AL subtypes via cytochemistry. However, ALP may help distinguish CD34+ AL from B and T cell lymphomas. FC‐based ALP analysis is not a reliable marker for CD34+ AL classification.

## 1 Introduction

Acute leukaemia (AL) is a rare, aggressive, and heterogeneous haematopoietic neoplasm that can occur in many species, including dogs, cats, horses and humans. The prognosis for canine AL remains poor, and standardised classification schemes in veterinary medicine are lacking [1, 2, 3, 4]. Broadly, AL is classified based on lineage commitment to either lymphoid or myeloid cell types—acute lymphoid leukaemia (ALL) or acute myeloid leukaemia (AML), respectively—although some cases lack defining morphologic or immunophenotypic features and are considered acute unclassifiable leukaemia (AUL). This distinction is clinically important because these subtypes may differ in therapeutic approach, with ALL often treated using CHOP‐ or COP‐based lymphoma chemotherapy protocols and AML managed using variations of CHOP, anthracyclines and/or cytarabine. However, responses in both ALL and AML are generally limited in duration and overall prognosis remains poor [5, 6]. Accurate classification also is essential for designing studies and evaluating novel therapies that may have lineage‐specific effects. In veterinary diagnostics, AL is distinguished using a combination of techniques, such as flow cytometry, immunocytochemistry and cytochemical staining [7, 8, 9, 10, 11, 12]. Currently, flow cytometry is considered the preferred method for subclassifying AL in veterinary patients, as it is cost‐effective and allows for the simultaneous assessment of multiple antigen markers. However, cytochemical stains, historically regarded as the gold standard for AL lineage determination, remain in routine use. Common cytochemical stains used in veterinary diagnostics for distinguishing haematopoietic tumours include peroxidase, Sudan Black B, naphthol AS‐D chloroacetate esterase, alpha‐naphthyl butyrate esterase, acid phosphatase and alkaline phosphatase (ALP), among others [13, 14].

ALP has historically been regarded as a cytochemical marker for the monocytic cell types in dogs; however, its presence is not entirely restricted to this lineage and has also been detected in other canine leukocytes, including eosinophils [15]. Additionally, ALP enzymatic activity is detectable in multiple tissue types and has been reported to be expressed in human pluripotent and embryonic stem cells [16]. Recent human studies have revealed that ALP is detectable in poorly differentiated acute leukaemias and has been proposed as a marker for leukaemic stem cells in human AML cases [17, 18]. Facklam and Kociba found ALP enzymatic activity to be detectable in 38% of canine lymphocytic leukaemia cases; however, this study did not utilise flow cytometry to subtype the leukaemias [14].

**FIGURE 1:**
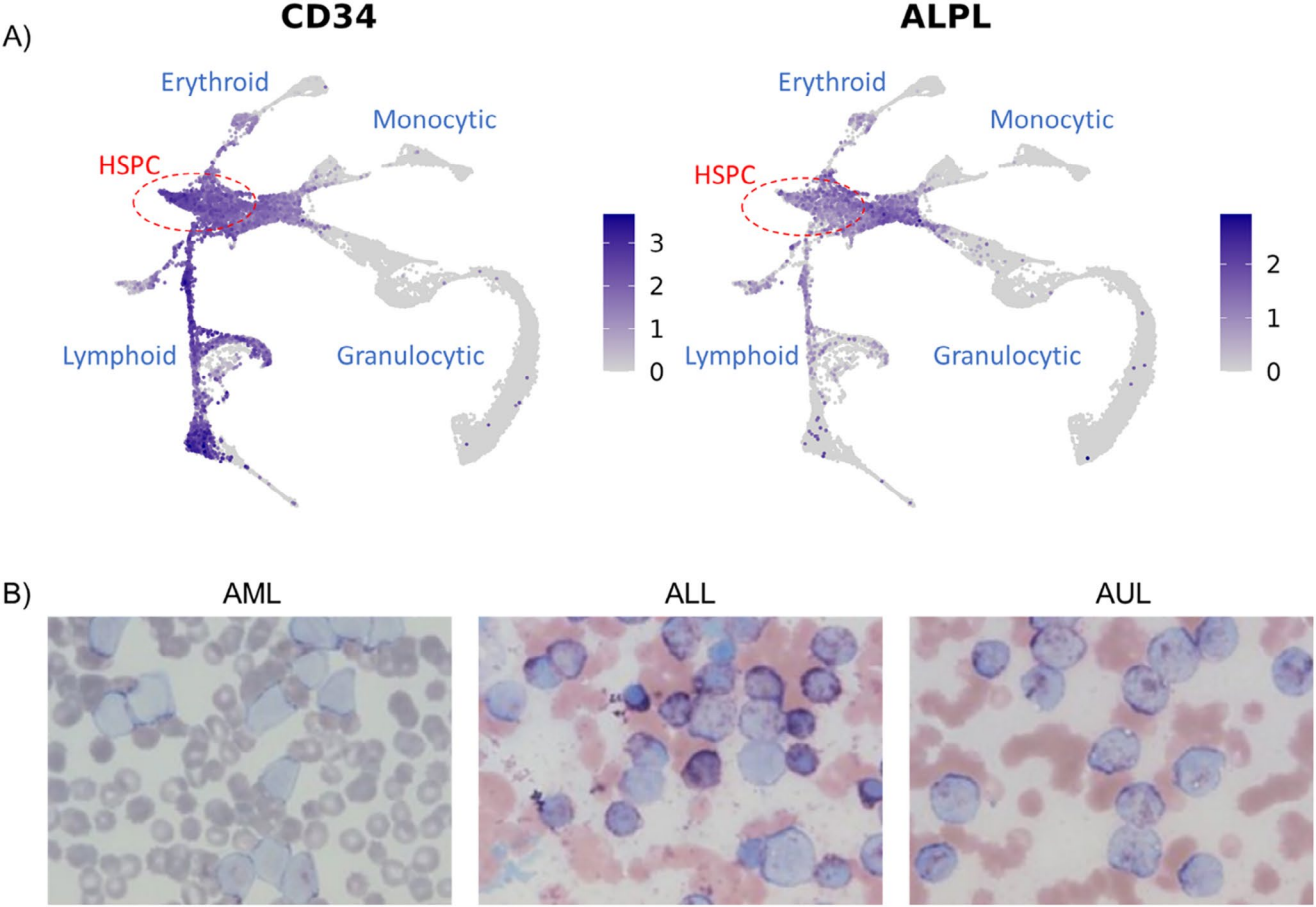
Overview of cytochemical staining and flow cytometric analysis among AL subtypes. (A) Feature plots highlighting the normalised gene expression *CD34* (left) and *ALPL* (right) gene expression in haematopoietic cell populations, demonstrating *ALPL* localization to a subset of CD34+ cell types [19]. Expression values represent normalised log‐transformed counts from single‐cell RNA‐sequencing data. Darker purple indicates higher expression, while grey represents low or absent expression. Datapoints are ordered to overlay cells with non‐zero expression above those with zero expression. Presumptive haematopoietic and progenitor cells (HSPC) are circled in red. The erythroid, lymphoid, monocytic and granulocytic branches are collectively identified. (B) Representative images of cytochemical staining for ALP enzymatic activity in AML, ALL, and AUL cases. Staining intensity and the proportion of ALP+ neoplastic cells varied across AL subtypes. From left to right: An AML case with weak cytochemical staining, followed by an ALL and an AUL case with strong cytochemical staining (100× objective, NBT/BCIP stain).

ALP has since been proposed as a useful marker for distinguishing canine acute myelomonocytic neoplasms from other AL subtypes [8]. In the work by Stokol et al., all 20 AML cases were considered ALP+ by cytochemical staining using a ≥ 3% positivity threshold, and 16 of those were considered CD34+ by flow cytometry [8]. Interestingly, 5 out of 12 T cell lymphoma/leukaemia cases were CD34+ and 4 of those were also considered ALP+ by cytochemical staining [8]. Previous transcriptomic profiling of canine bone marrow haematopoietic cells performed by our group demonstrated that ALP gene expression (*ALPL*) is predominantly localised to haematopoietic stem and progenitor cell populations that co‐express CD34 (Figure 1A) [19]. Given these findings, we sought to evaluate ALP enzymatic activity across CD34+ AL subtypes (i.e., AML, ALL and AUL) and assess whether this activity could aid in their distinction and additionally identify early progenitor cell types.

This study consisted of two components. First, we conducted a prospective analysis to evaluate ALP enzymatic activity in CD34+ AL subtypes using both cytochemical staining and flow cytometry, and determine the utility of ALP enzymatic activity in differentiating myeloid from lymphoid CD34+ AL. In the second component, we performed a retrospective analysis of archived cytologic samples to assess ALP enzymatic activity in flow cytometry–confirmed CD34+ ALs as well as B‐cell and T‐cell lymphomas.

## 2 Methods

### 2.1 Case Selection

An overview of our study design and methodology is provided in Figure S1. No samples were solicited for this study. Peripheral blood samples from 10 normal (healthy) control dogs without evidence of haematopoietic neoplasia were obtained as discarded materials from Colorado State University‘s (CSU) Clinical Pathology Laboratory. Control dogs were considered healthy if they had no clinical or laboratory evidence of haematopoietic neoplasia or other systemic disease documented in their medical records. Per institutional policies, these samples were exempt from IRB approval. Peripheral blood samples from dogs with suspected lymphoproliferative disorders were originally submitted for immunophenotyping by flow cytometry to the Clinical Hematopathology laboratory between June 8, 2023, and August 11, 2023. Dogs were included if flow cytometry confirmed a diagnosis of CD34^+^ acute leukaemia (AL, *n* = 64) or B cell chronic lymphocytic leukaemia (BCLL, *n* = 10) based on previously established criteria [7, 20]. Dogs were excluded if samples had insufficient material for ALP staining.

To assess ALP staining in tissue samples, the Clinical Hematopathology database was queried for retrospective cytologic specimens with concurrent flow cytometry data and cytology slides available. The search was restricted to cases from 1 January 2018, to 30 September 2023. Sixty‐seven cases were randomly selected, including 18 B cell lymphomas, 22 T cell lymphomas of various immunophenotypes, and an additional 27 CD34+ AL cases. Samples were not restricted by age, sex or breed. Samples were excluded if cytology slides were not retrievable. Signalment, hematologic parameters, and clinical data were extracted from laboratory submission forms and medical records. Complete demographic details for all cases are provided in Table S1 (hematologic cases) and Table S2 (tissue cases).

### 2.2 Cell Line Validation Statement

No cell lines were used in the current study.

### 2.3 Complete Blood Counts (CBC)

Peripheral blood CBCs were either provided by the clinic prior to submission or performed at the CSU's Clinical Pathology Laboratory. The haematology analyser information (manufacturer, model, and reference intervals) for in‐house clinic CBC and non‐CSU CBCs was not available. CBCs performed at CSU were analysed on an Advia 120 (Siemens AG, Munich, Germany), and manual blood films were reviewed by a pathologist. Automated platelet counts were abstracted from the CBC, but not all cases had blood film reviews at the time of performing CBC to assess platelet clumping.

### 2.4 Cytology and Cytochemical Staining

Blood smears for cytologic evaluation and ALP staining were prepared from the same EDTA samples after shipment and arrival at the Clinical Hematopathology Laboratory. ALP staining for haematology cases was performed within 3 days of sample receipt. All slides were evaluated in a blinded manner with respect to diagnosis. Samples were stained with a modified Wright‐Giemsa stain and evaluated by a board‐certified clinical pathologist (RAH). Subsequently, selected areas across multiple fields of the Modified‐Wright Giemsa–stained slides, chosen for optimal cellular preservation and representation, were stained with Phosphatase Substrate (NBT/BCIP, SeraCare) for 1 h, following previously published protocols [21]. A 200‐cell differential was performed on the slides to determine the proportion of ALP+ and ALP‐ presumptive neoplastic cells. After ALP staining, presumptive neoplastic cells were identified by correlating cytochemical staining patterns with underlying cytologic features consistent with malignancy, including cell morphology, size, and nuclear characteristics, while normal leukocytes were distinguished based on their expected morphology. Cases were considered ALP+ if they met the previously proposed cutoff of 3% [8, 13]. This threshold cutoff was further supported by ROC analysis (see below). ALP staining characteristics were subjectively categorised as weak or strong based on the intensity of staining. Defined staining intensities aligned with previously proposed criteria: weak = light cytoplasmic stippling or granularity; strong = moderate to marked cytoplasmic stippling [8].

### 2.5 Flow Cytometry

Routine immunophenotyping was performed on all fresh blood samples as previously described [22]. Samples were acquired over a period during which the diagnostic staining panel was modified. At the Clinical Hematopathology laboratory, samples submitted between 7 March 2017, and 13 November 2019, were stained with antibody panel 1; and samples submitted after 13 November 2019, were stained with antibody panel 2. The two panels are provided in Table S3 and all samples were ran on a 3 laser Coulter Gallios instrument (Beckman Coulter Inc., Brea, California). The panel does not detect intracellular antigens. The diagnosis of acute leukaemia came from a combination of clinical history, available CBC laboratory data, and meeting one or both of the following flow cytometry immunophenotyping criteria: (1) > 10% of total leukocytes expressed the antigen marker CD34; or (2) > 1000 cells/μL expressed CD34 by flow cytometry. CD34+ AL cases were further classified into lymphoid (ALL), myeloid (AML) or unclassifiable (AUL) using previously published criteria [7]. Briefly, ALL cases were diagnosed if > 13% of neoplastic cells expressed CD5, AML cases were diagnosed if > 3.0% of suspected neoplastic cells were CD14+ MHCII− or if > 18% of suspected neoplastic cells were CD18+ MHCII− and AUL cases were diagnosed if they lacked expression of any lineage‐specific surface antigen. Flow cytometric results for classifying CD34+ AL cases by subtype are provided in Table S4 for haematology (H) cases (H1–H64) and tissue (T) cases (T1–T27). Cases were identified as B cell CLL if they had (a) > 5 × 10^3^ lymphocytes/μL on CBC, (b) > 60% of the lymphocytes were CD21+ B cells and (c) B cells were considered small‐sized, defined by B cell: neutrophil median forward scatter < 0.6 [20].

Samples from tissue were characterised via flow cytometry using the same antibody panel as mentioned above. CD34+ AL subtypes were classified as described above. Lymphoma samples included: Two cases small cell B cell lymphoma with > 80% small CD21+ B cells [23]; 16 cases large cell B cell lymphoma with CD21+ B cells accounting for > 60% of the large cells [24]; 14 cases CD4+ T cell lymphoma with a homogeneous expansion of CD4+ CD45+ T cells expressing low levels of class II MHC [25]; 2 cases CD4‐CD8‐ T cell lymphoma with a homogeneous expansion of aberrant CD4‐CD8‐CD45+ T cells [26]; and 6 cases T zone lymphoma with > 20% small CD5+ CD45− T cells expressing high levels of class II MHC [22].

For ALP assessment via flow cytometry, the normal control samples, B cell CLL, and CD34+ AL samples were stained for surface antigens, including CD5, CD4 and CD34, and using the ALP live stain (Life Technologies Catalogue: A14353). The ALP live stain was performed following the manufacturer's protocol with a few modifications. Specifically, cells were incubated in a 1:500 dilution of ALP live stain prepared in flow cytometry buffer for 20 min at room temperature in the dark. Post‐staining, samples were washed with flow buffer and then subsequently ran on a 3‐laser Coulter Gallios instrument (Beckman Coulter Inc., Brea California). Flow cytometry data was analysed using Kaluza Analysis Software (Beckman Coulter Inc.). A fluorescence minus one (FMO) control for ALP was included in the flow cytometry experimental design (Table S3). The ALP gating strategy consisted of the following: Post‐gating for singlets (as determined by forward scatter time‐of‐flight vs. forward scatter area) and live cells (propidium iodide negative), we focused on detection of ALP enzymatic activity. Neutrophils were noted to be strongly positive for ALP activity (Figure S2A); therefore, CD4+ CD5− cells were selectively gated out to minimise the inclusion of non‐neoplastic cells and focus on putative leukaemic populations. Subsequently, we determined the proportion of CD34+ ALP+, CD34− ALP+, CD34+ ALP− and CD34− ALP− cells remaining post‐neutrophil exclusion (Table S1). Additionally, median fluorescence intensity (MFI) of ALP+ populations was recorded, stratified by CD34 expression status (CD34+ ALP+ vs. CD34− ALP+).

### 2.6 Statistical Analysis

Statistical significance was set at *p* < 0.05 for all analyses. For continuous variables, normality was assessed using the Shapiro–Wilk test. For comparisons across multiple groups, either a one‐way ANOVA or Kruskal–Wallis test was used if variables passed or did not pass normality, respectively. For pairwise comparisons between CD34+ AL subtypes, unpaired t‐tests were used when normality assumptions were met. For categorical and ordinal variables, Fisher's exact test or Chi‐square test was applied for comparisons. Additionally, given that the primary focus of our analysis was to compare ALP enzymatic activity in AML against other CD34+ AL subtypes, ALL and AUL cases were combined in categorical and ordinal analyses to increase statistical power. To assess the correlation between the percentage of ALP+ cells detected by flow cytometry and those estimated by cytochemical staining, Spearman's rank correlation coefficient was calculated. ROC analysis was performed using ALP% as determined by cytochemical staining, to evaluate its diagnostic performance in distinguishing CD34+ AL from B cell CLL (in blood samples) and from lymphoma (in tissue samples). Additional ROC analyses were conducted to assess whether cytochemical ALP% could differentiate AML from ALL/AUL. ROC analysis was not performed using flow cytometry‐based ALP detection due to inconsistencies in performance across cases. All statistical analyses were conducted using GraphPad Prism version 8 (GraphPad Software Inc.).

## 3 Results

### 3.1 Study Population

EDTA peripheral blood was available from 84 dogs, including 10 normal controls, 64 CD34+ AL cases (31 AML, 12 ALL and 21 AUL), and 10 B cell CLL cases. An overview of the cohort is provided in Table 1. The control group included five female spayed, four male castrated, and one intact male dog, with ages ranging from 1.5 to 13.8 years (median 7.3 years). An overview of the signalment data, cytochemical staining results and flow cytometric data for each hematologic case is provided in Table S1.

**TABLE 1:** Overview of entire cohort.

		Number affected (% of group) or median (IQR)										
		Hematologic ALP evaluation (*n* = 84)					Tissue ALP evaluation (*n* = 67)					
		Normal	B cell CLL	AML	ALL	AUL	B cell lymphoma^b^	Aggressive T cell lymphoma^c^	T zone lymphoma	AML	ALL	AUL
Group total (*n*)		10	10	31	12	21	18	16	6	18	5	4
Sex	Intact female	0 (0.0%)	0 (0.0%)	0 (0.0%)	1 (8.3%)	1 (4.8%)	1 (5.6%)	1 (6.3%)	0 (0%)	1 (5.6%)	0 (0%)	0 (0%)
	Spayed female	5 (50.0%)	7 (70.0%)	11 (35.5%)	5 (41.7%)	9 (42.9%)	6 (33.3%)	5 (31.3%)	3 (50.0%)	9 (50.0%)	2 (40.0%)	3 (75.0%)
	Intact male	1 (10.0%)	0 (0.0%)	1 (3.2%)	1 (8.3%)	1 (4.8%)	1 (5.6%)	1 (6.3%)	1 (16.7%)	1 (5.6%)	0 (0%)	0 (0%)
	Neutered male	4 (40.0%)	3 (30.0%)	19 (61.3%)	5 (41.7%)	10 (47.6%)	10 (55.6%)	9 (56.3%)	2 (33.3%)	7 (38.9%)	3 (60.0%)	1 (25.0%)
Age		7.3 (4.5–11.5)	12.9 (10.9–14.1)	9.0 (6.5–9.5)	7.8 (5.1–9.1)	10.0 (3.5–10.4)	9.4 (7.9–11.0)	7.2 (5.5–9.0)	10.3 (8.3–11.9)	9.0 (8.0–10.2)	9.0 (8.0–10.0)	7.8 (6.7–8.9)
Hematologic data (when available)	WBC (cells/μL)	8350 (6847–9750)	40 600 (29 500–62 817)	60 800 (19 250–116 400)	140 260 (54 063–238 200)	148 800 (63 160–183 200)	—	—	—	—	—	—
	HCT (%)	47.0 (43.8–48.8)	42.5 (33.8–49.3)	30.0 (25.5–34.5)	30.5 (24.5–36.5)	25.0 (23.0–31.0)	—	—	—	—	—	—
PLT (cells/μL)^a^		325 500 (247 500–443 500)	263 000 (186 500–430 000)	75 000 (56 250–126 000)	71 000 (37 750–77 250)	52 000 (22 000–84 000)	—	—	—	—	—	—
Tissue site	Visceral LN	—	—	—	—	—	0 (0%)	0 (0%)	0 (0%)	2 (11.1%)	0 (0%)	0 (0%)
	Peripheral LN	—	—	—	—	—	16 (88.9%)	15 (93.8%)	6 (100%)	14 (77.8%)	4 (80.0%)	2 (50.0%)
	Mediastinal mass	—	—	—	—	—	1 (5.6%)	1 (6.3%)	0 (0%)	0 (0%)	1 (20.0%)	2 (50.0%)
	Effusion	—	—	—	—	—	1 (5.6%)	0 (0%)	0 (0%)	1 (5.6%)	0 (0%)	0 (0%)
	Liver	—	—	—	—	—	0 (0%)	1 (6.3%)	0 (0%)	0 (0%)	0 (0%)	0 (0%)
	Spleen	—	—	—	—	—	0 (0%)	0 (0%)	0 (0%)	1 (5.6%)	0 (0%)	0 (0%)

Abbreviations: ALL, acute lymphoid leukaemia; ALP, alkaline phosphatase; AML, acute myeloid leukaemia; AUL, acute unclassified leukaemia; B cell CLL, B cell chronic lymphocytic leukaemia; HCT, haematocrit; LN, lymph node; PLT, platelet count; WBC, white blood cell.

^a^ PLT counts included regardless of whether clumping was identified or not.

^b^ B cell lymphoma group includes both large and small B cell subtypes.

^c^ Aggressive T cell lymphoma includes CD4+ and CD4‐CD8‐ subtypes.

For cytologic samples from tissue aspirates, we analysed specimens from 56 peripheral lymph nodes, 2 visceral lymph nodes, 5 mediastinal masses, 1 liver and 1 spleen. Additionally, 2 effusion samples were also analysed within this group. Of the 67 total cases, 27 were CD34+ AL and 40 were various subtypes of lymphoma, including 14 CD4+ T cell lymphoma, 2 CD4− CD8− T cell lymphoma, 6 T zone lymphoma, 2 small cell B cell lymphoma, and 16 large cell B cell lymphoma. The CD4+ T cell lymphomas and the 2 CD4− CD8− T cell lymphomas were collectively categorised as ‘Aggressive T cell lymphoma’ when compared to T zone lymphoma cases. Among the CD34+ AL cases, 4 were AUL, 18 were AML and 5 were ALL. An overview of the signalment data, associated metadata for the site sampled, and flow cytometric data for each tissue case are provided in Table S2.

### 3.2 Hematologic Parameters in Peripheral Blood Cases and Sample Site Distribution in Tissue Cases

Hematologic parameters were available for most hematologic cases and are summarised in Table 1. Among CD34+ AL cases, WBC counts varied widely, with median values highest in ALL and AUL. Anaemia and thrombocytopenia were common among CD34+ AL cases. However, platelet counts should be interpreted with caution because manual smear quantification was not performed, some samples had platelet clumping or unknown clumping status, and cytoplasmic fragments may have interfered with automated platelet enumeration (Table S1).

For tissue‐based ALP evaluation, specimen collection sites for these cases varied and are summarised in Table 1 and complete details are provided in Table S2. Most lymphoma cases were derived from peripheral lymph nodes (*n* = 17/18 for B cell lymphoma, *n* = 13/14 for CD4+ T cell lymphoma, *n* = 1/2 for CD4‐CD8‐ T cell lymphoma and *n* = 6/6 for T zone lymphomas). Most CD34+ AL cases also originated from peripheral lymph nodes (*n* = 14/18 AML, *n* = 4/5 ALL, *n* = 2/4 AUL), while additional cases were obtained from visceral lymph nodes (*n* = 2/18 AML), mediastinal masses (*n* = 1/5 ALL, *n* = 2/4 AUL), effusions (*n* = 1/18 AML) and the spleen (*n* = 1/18 AML). The original cytologic diagnosis was extrapolated from the pathology reports associated with each case (Table S2). Notably, 21 of 27 AL cases (77.8%) were originally diagnosed as ‘lymphoma’ or ‘possible lymphoma’.

When flow cytometric data from blood and tissue samples were combined, the proportion of CD34+ cells was significantly lower in AML cases compared to AUL and ALL cases (unpaired *t*‐test *p* < 0.01). However, no significant differences were observed between AUL and ALL (unpaired *t*‐test *p* > 0.05). Additionally, the percentage of CD34+ cells did not significantly differ between blood and tissue samples within any AL subtype (Mann–Whitney test *p* > 0.05).

### 3.3 Cytochemical Detection of ALP Enzymatic Activity Cannot Distinguish CD34+ Acute Leukaemia Subtypes

**FIGURE 2:**
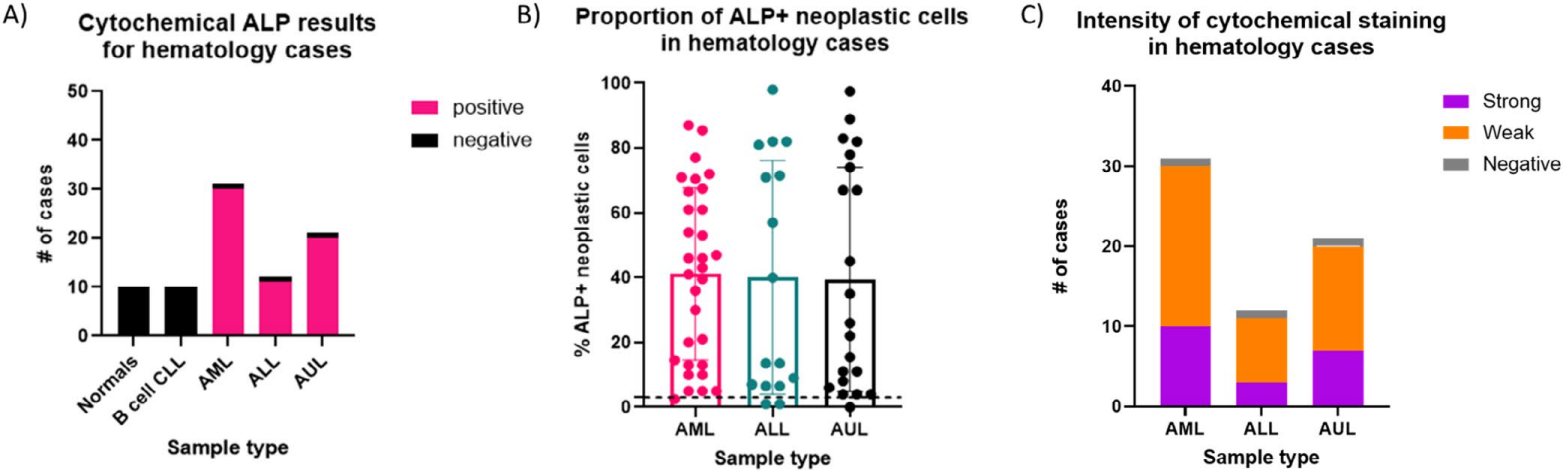
Cytochemical staining results for ALP enzymatic activity in haematology cases. (A) Bar plot displaying the proportion of ALP+ (≥ 3% ALP+ cells by cytochemical staining) and ALP− cases across normal controls, B cell CLL, and CD34+ AL subtypes. (B) Box plot illustrating the proportion of ALP+ neoplastic cells identified by cytochemical staining. Dashed line represents the 3% cutoff for determining ALP+ vs. ALP− cases. No significant difference was observed between CD34+ AL subtypes (Kruskal–Wallis test *p* > 0.05). (C) Stacked bar plot showing ALP staining intensity across AL subtypes. When ALL and AUL cases were combined and compared to AML, no statistical difference was observed between the i of cytochemical staining (Fisher's exact test *p* > 0.05).

Cytochemical staining for ALP enzymatic activity in our normal control samples (*n* = 10) yielded expected results, in that eosinophils were considered positive, and neutrophils were considered negative. None of the neoplastic B cell CLL cases (*n* = 10) were considered positive, which is consistent with previous reports [8]. In contrast, ALP enzymatic activity was detected in most of the CD34+ AL cases (*n* = 61/64, 95.3%) (Figure 2A).

To assess diagnostic performance, we performed ROC analysis of ALP% to differentiate AL from B cell CLL using blood samples, which showed high sensitivity and specificity (area under curve (AUC) = 0.99) (Figure S3A and Table S5). Using the previously published 3% cutoff for ALP^+^ (putative) neoplastic cells (sensitivity: 95.3% [95% Confidence Interval (CI): 87.1%–98.7%], specificity: 100% [95% CI: 72.3%–100%]), the majority of cases were classified as ALP^+^, including 96.8% of AML (30/31), 91.7% of ALL (11/12) and 95.2% of AUL (20/21) cases. Three cases (one AML, one ALL and one AUL) fell below this 3% threshold and were therefore considered negative for ALP enzymatic activity. Based on these findings and consistency with published criteria, the 3% ALP+ threshold was retained for classification purposes in this study [8, 13].

We noted that the proportion of ALP+ (putative) neoplastic cells in CD34+ AL cases varied widely, ranging from 0% to 98.0%, with staining intensities classified as weak or strong. We did not find a statistical difference in the proportion of ALP+ cells detected across CD34+ AL subtypes (Kruskal–Wallis test *p* > 0.05, Figure 2B). Furthermore, ROC analysis yielded limited discriminatory value between CD34+ AL subtypes (Figure S3B and Table S6, AUC = 0.52). Additionally, staining intensities across all CD34+ AL subsets were considered variable (Figure 1B), making it difficult to reliably distinguish between subtypes based on the staining intensity of ALP enzymatic activity alone in this cohort (Fisher's exact *p* > 0.05, Figure 2C).

### 3.4 Flow Cytometric Detection of ALP Enzymatic Activity Is Discordant With Cytochemical Analysis

Next, we evaluated whether ALP enzymatic activity measured by flow cytometry could distinguish CD34+ AL from normal and B cell CLL samples and between CD34+ AL subtypes. Representative images of our gating strategy for defining the cell populations of interest are provided in Figure S2. Flow cytometric data were available in a subset of hematologic cases (Table S1). We found that, in contrast to the cytochemical stain, canine neutrophils had ALP enzymatic activity when measured with this reagent. In addition, we noted that even after excluding neutrophils from downstream analysis, variability in ALP enzymatic activity persisted among CD34− negative cells, including within our controls (*n* = 7) and B cell CLL cases (*n* = 3). Although normal and B cell CLL samples did not have detectable CD34+ cell populations, the proportion of CD34^−^ALP^+^ cells varied widely (4.47%–67.4% post‐neutrophil exclusion), even after removal of neutrophils from the analysis.

Despite finding that ALP live‐stain reagent was detected in various cell types, we wanted to assess whether this approach could distinguish CD34+ AL cases. Therefore, we focused downstream comparisons on CD34+ cells, as these are known neoplastic populations in AL. We noted that the correlation between the percentage of CD34+ ALP+ cells detected by flow cytometry and the percentage of ALP+ (putative) neoplastic cells detected by cytochemical staining was considered weak (Spearman's rho = 0.25, Figure S4A). No statistically significant differences were detected between the proportion of CD34+ ALP+ cells in CD34+ AL subtypes (ANOVA *p* > 0.05, Figure S4B). Similarly, ALP MFI on CD34+ ALP+ cells did not significantly differ across subtypes (ANOVA *p* > 0.05, Figure S4C). In summary, the ALP reagent detected by flow cytometry lacked specificity for distinguishing CD34+ AL subtypes, and we also noted that it was present in normal leukocytes and other haematopoietic tumours. Given the discordant results with cytochemistry, it remains unclear whether the reagent is accurately detecting ALP enzymatic activity in canine leukocytes.

### 3.5 Cytochemical Staining for ALP Enzymatic Activity May Aid in Distinguishing CD34+ AL From CD34‐ Haematopoietic Neoplasms

**FIGURE 3:**
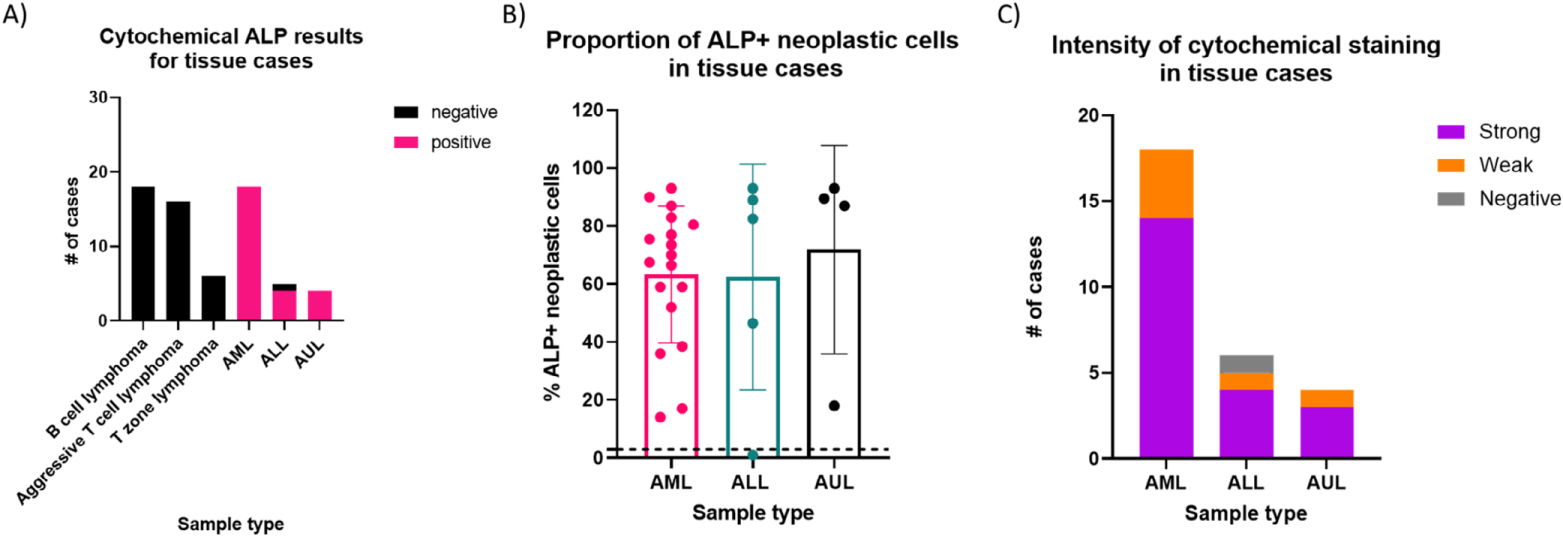
Cytochemical staining results for ALP enzymatic activity in tissue samples. (A) Bar plot showing the distribution of ALP+ (≥ 3% ALP+ cells by cytochemical staining) and ALP− cases across B cell lymphoma, aggressive T cell lymphoma (CD4+ and CD4− CD8− subtypes), T zone lymphoma, and CD34+ acute leukaemia (AL) subtypes. When B cell and T cell lymphoma cases were combined and compared with CD34+ AL subtypes, a Fisher's exact test revealed a statistically significant difference (*p* < 0.0001). Additionally, when ALL and AUL cases were combined and ALP+ vs. ALP− cases were compared to AML, no statistically significant difference was observed (Fisher's exact test *p* > 0.05). (B) Box plot illustrating the proportion of presumptive ALP+ neoplastic cells identified in tissue samples. No statistically significant difference was detected between AL subtypes (Kruskal–Wallis test *p* > 0.05). (C) Stacked bar plot representing the cytochemical staining intensity of ALP+ cases across CD34+ AL subtypes. When ALL and AUL cases were combined and compared to AML, no statistically significant difference was observed (Fisher's exact test *p* > 0.05).

Given that CD34+ AL in dogs often involves peripheral lymph nodes or organs, we hypothesised that ALP enzymatic activity could help distinguish CD34+ AL from other haematopoietic neoplasms, such as nodal B cell and T cell lymphomas, that are commonly diagnosed via cytology [27]. Therefore, we retrospectively evaluated ALP enzymatic activity in cytology samples from tissue or effusion specimens that had concurrent flow cytometry immunophenotyping data. To address the potential impact of sample archival time on ALP detection, we evaluated the relationship between time since staining (range: 0.01–5.61 years, median: 1.1 years, interquartile range: 0.19–2.31 years). No significant correlation was observed between archival time and ALP% in ALP+ cases (Spearman's *ρ* = −0.18, *p* = 0.39), suggesting stable enzymatic activity across the time frame evaluated. ROC analysis on tissue samples to assess the discriminatory performance of ALP% between CD34+ AL and lymphoma revealed an AUC = 0.99 (Figure S3C and Table S7). Two thresholds demonstrated excellent performance in tissue samples: a 1% cutoff yielded 96.3% sensitivity (95% CI: 81.7%–99.8%) and 97.5% specificity (95% CI: 87.1%–99.9%), while an 8% cutoff provided 96.3% sensitivity (95% CI: 81.7%–99.8%) and 100% specificity (95% CI: 91.2%–100%). However, to maintain consistency with the hematologic cases aforementioned, a 3% cutoff of %ALP+ (putative) neoplastic cells was applied to the tissue samples. Using this 3% ALP+ threshold, all T cell lymphoma (*n* = 22) and B cell lymphoma (*n* = 18) cases were ALP negative by cytochemical staining (Table 2; Figure 3A). In contrast, 96.3% (*n* = 26/27) of CD34+ AL tissue samples exhibited ALP positivity, with ALP+ (putative) neoplastic cell percentages ranging from 14% to 93% (median: 74.5%, Figure 3B). Similar to CD34+ AL blood samples, no statistically significant differences were observed between CD34+ AL subtypes in either ALP% (Kruskal–Wallis *p* > 0.05) or staining intensity (Fisher's exact *p* > 0.05; Figure 3B,C). Lastly, ROC analysis comparing AML vs. ALL/AUL in tissue revealed limited discriminatory power (Figure S3D and Table S8, AUC = 0.63).

**TABLE 2:** Summary of cytochemical and flow cytometric ALP results.

	Tumour type	Number affected (% of group) or median (IQR)		
		Cytochemical		
		(*n*)	ALP+ cases	% neoplastic cells ALP+
Hematologic ALP evaluation (*n* = 85)	Normal	10	0 (0%)	—
	B cell CLL	10	0 (0%)	0
	AML	31	30 (96.8%)	43.0 (13.8–63.8)
	ALL	12	11 (91.7%)	48.6 (8.5–73.9)
	AUL	21	20 (95.2%)	26 (8.0–74.0)
Tissue ALP evaluation (*n* = 67)	B cell Lymphoma	18	0 (0%)	0
	Aggressive T cell lymphoma^a^	16	0 (0%)	0
	T zone lymphoma	6	0 (0%)	0
	AML	18	18 (100%)	68.8 (53.8–79.6)
	ALL	5	4 (80%)	86.0 (73.9–90.0)
	AUL	4	4 (100%)	88.3 (69.8–90.4)

Abbreviations: ALL, acute lymphoid leukaemia; ALP, alkaline phosphatase; AML, acute myeloid leukaemia; AUL, acute unclassified leukaemia; B cell CLL, B cell Chronic Lymphocytic Leukaemia.

^a^ Aggressive T cell lymphoma includes CD4+ and CD4− CD8− subtypes.

## 4 Discussion

In this study, we evaluated ALP enzymatic activity as a potential marker for distinguishing CD34+ AL subtypes from other CD34‐negative haematopoietic neoplasms using both cytochemical staining and flow cytometry methodologies. Our results demonstrate that ALP enzymatic activity is consistently detected in CD34+ AL using cytochemical staining, but was unable to differentiate AML, ALL, or AUL cases. After confirming that ALP enzymatic activity was present in most CD34+ AL haematology cases, we expanded our analysis to determine whether ALP detection could distinguish these tumours from other CD34‐negative haematopoietic malignancies, such as nodal B cell and T cell lymphomas. In this cohort, ALP enzymatic activity was a distinguishing feature of CD34+ AL compared to B cell and T cell lymphomas. This distinction is clinically relevant, as 77.8% (21/27) of CD34+ AL cases in our study were originally classified on cytology as ‘lymphoma’ or ‘possible lymphoma’, highlighting the diagnostic challenges when AL presents in tissues. Overall, these findings suggest that ALP detection via cytochemistry, while not useful for subclassifying CD34+ AL, may serve as a practical diagnostic tool to differentiate precursor haematopoietic neoplasms from more differentiated lymphoid malignancies in cytologic specimens. Although cytochemical ALP staining did not differentiate between AML and ALL subtypes, the ability to distinguish CD34^+^ acute leukaemias from other haematopoietic neoplasms provides a foundation for improved classification schemes in veterinary medicine. Enhanced classification not only supports accurate diagnosis but also lays the groundwork for future studies evaluating targeted therapies and novel treatment protocols, where lineage‐specific strategies may emerge.

The proportion of ALP+ cells and the strength of cytochemical staining were not statistically different between AML, ALL, and AUL. Our findings contrast with previous studies, which have reported ALP enzymatic activity associated with specific AL subtypes, particularly in AML [8]. Traditionally, ALP enzymatic activity has been suggested as a marker of monocytic or myeloid lineage [13]. However, our gene expression data highlighted that *CD34* and *ALPL* transcripts were concurrently expressed in canine haematopoietic stem and progenitor cells. Previous studies in humans have shown that ALP enzymatic activity can be found in pluripotent stem cells and CD34+ leukaemic cells [16, 18, 28]. It is possible that ALP enzymatic activity may originate in haematopoietic stem and progenitor cells, persist in myeloid‐committed cells, and become downregulated in lymphoid‐committed cells in dogs. Regardless, this may have clinical relevance as a previous study reported that human AML patients with > 12% ALP+ leukaemic blasts had shorter overall survival [17]. A similar paradigm may exist in veterinary patients, warranting further investigation into the functional role and clinical significance of ALP activity in canine CD34+ AL.

Flow cytometric detection of ALP using the reagents in this study produced results that were inconsistent with cytochemical staining. Specifically, ALP enzymatic activity was detected across a variety of normal peripheral blood leukocytes and B cell CLL cases, raising concerns about nonspecific staining. Notably, we also observed that canine neutrophils exhibited ALP positivity by flow cytometry, despite the same blood samples having ALP‐negative neutrophils by cytochemical staining and previous studies indicating canine neutrophils are ALP‐negative [15]. It is possible that the observed findings represent nonspecific staining or off‐target effects in canine leukocytes. At this point, these inconsistencies highlight limitations in using flow cytometry for ALP detection in canine leukaemia and suggest that cytochemical staining remains the more reliable approach. Based on these observations, we recommend that future studies evaluating ALP in canine haematopoietic neoplasms prioritise cytochemical staining methods, which demonstrated greater specificity in our analysis. The nonspecific reactivity detected by the live‐cell ALP reagent in flow cytometry underscores the need for further validation of this approach before it can be reliably applied in research or diagnostic settings.

Several limitations must be acknowledged. First, not all subtypes of lymphoma/leukaemia were tested, so assessing ALP enzymatic activity in a larger cohort of non‐AL and CD34‐negative AL cases could be useful to determine the specificity of ALP positivity for CD34+ AL. We also did not test ALP enzymatic activity in other non‐lymphoid forms of round cell neoplasia, which may be differentials in cytology specimens diagnosed as ‘round cell tumour’. Additionally, the retrospective nature of the study and reliance on banked samples introduce potential variability in sample handling and quality. We acknowledge that archival storage of retrospective samples is a potential limitation. While our study included cytologic specimens stored for up to 5 years, we did not identify a significant correlation between sample archival time and ALP% by cytochemical staining. Additionally, we note that prior studies have successfully performed cytochemical ALP staining on retrospective samples spanning multiple years [21]. Nevertheless, the impact of long‐term storage on enzyme stability and staining performance remains an important pre‐analytical consideration, and a formal prospective study is warranted to rigorously assess this variable. Lastly, the interpretation of cytochemical staining is also subject to a degree of observer bias, given its semi‐quantitative and subjective nature. Despite these limitations, our findings support the need for further investigation into the biological and clinical relevance of ALP enzymatic activity in canine CD34+ AL.

In summary, our findings suggest that ALP positivity, as determined by cytochemical staining, is broadly associated with CD34+ AL cases and is not limited to AML. While ALP does not differentiate between CD34+ AL subtypes, its near‐universal detection in CD34+ AL, but absence in lymphomas suggests that it may serve as a useful marker for distinguishing precursor haematopoietic neoplasms from more differentiated hematologic malignancies in cytologic specimens. Given the ease of cytochemical ALP staining, its incorporation into diagnostic workflows could aid in the rapid classification of haematopoietic neoplasms when immunophenotyping is pending or cannot be performed and at least inform clinical decisions. Future studies should aim to expand the sample size and explore the mechanistic basis of ALP enzymatic activity among CD34+ acute leukaemias.

## Ethics Statement

No samples were solicited for this study. The samples utilised in this study were obtained as discarded materials from either the Clinical Pathology and/or Clinical Hematopathology Laboratories and, per institutional policies, were considered exempt from IRB approval.

## Conflicts of Interest

The authors declare no conflicts of interest.

## Supporting information


**Figure S1:** Overview of methodology used in this study. (A) Prospective evaluation of ALP enzymatic activity in peripheral blood samples (normal, B cell CLL, CD34+ AL) from cases submitted to the Clinical Hematopathology Laboratory. Red blood cells were lysed, and remaining leukocytes were incubated with surface antibodies (Table S1) and ALP reagent for 20 min at room temperature. Cells were then analysed by flow cytometry to assess ALP expression, and cytologic evaluation was performed following staining with NBT/BCIP substrate solution for 1 h. (B) Retrospective evaluation of ALP enzymatic activity in tissue and effusion samples. Cytologic specimens with available flow cytometric diagnosis were identified, and ALP activity was evaluated using the NBT/BCIP staining method for 1 h, followed by microscopic analysis. Figure created in Biorender.com.

**Figure S2:** Representative flow cytometric plots showing ALP expression in a normal control sample (A), a B cell CLL case (B), an AUL case (C) and an AML case (D). Each panel illustrates the gating strategy, including the exclusion of identifiable neutrophils (outlined in red) and the use of fluorescence‐minus‐one (FMO) controls (rightmost plots). (A) Topmost flow cytometry plots demonstrate ALP activity in neutrophils, which were excluded from downstream analysis. Despite the exclusion of neutrophils, ALP activity was detected in other peripheral blood leukocytes and across multiple sample types. Notably, CD34^−^ ALP^+^ cells (highlighted in green) were observed, suggesting a broader distribution of ALP expression beyond neutrophils.

**Figure S3:** Receiver operating characteristic (ROC) curves of ALP% determined by cytochemical staining for distinguishing acute leukaemia (AL) and acute myeloid leukaemia (AML) from other hematologic malignancies in blood and tissue samples. (A) Comparison of AL vs. B cell CLL (BCLL) in blood cases. (B) Comparison of AML vs. ALL/AUL in blood cases. (C) Comparison of AL vs. lymphoma using tissue samples. (D) Comparison of AML vs. ALL/AUL using tissue samples. Area under the curve (AUC) values are shown in each panel.

**Figure S4:** Flow cytometric analysis of ALP expression in CD34+ AL cases. (A) Correlation between the percentage of ALP+ neoplastic cells identified by cytochemical staining and the percentage of CD34 + ALP+ cells detected by flow cytometry (Spearman's rho = 0.25). (B) Box plot comparing the proportion of CD34 + ALP+ cells between haematology cases. No statistically significant difference in the proportion of CD34 + ALP+ cells was observed between CD34+ AL subtypes (ANOVA *p* > 0.05). (C) Box plot highlighting the MFI of ALP on CD34 + ALP+ cells. No statistical difference was observed between CD34+ AL subtypes (ANOVA *p* > 0.05).

**Data S1:** vco70024‐sup‐0006‐Tables.xlsx.

## Data Availability

The data that supports the findings of this study is available in the Supporting Information of this article.
