# Supplementary figures and images for: Utility of Cytochemical and Flow Cytometry Detection of Alkaline Phosphatase for Differential Diagnosis of CD34+ Acute Leukaemia in Canines
Source: Vet Comp Oncol. 2025 Oct 25;24(1):41–50. doi: 10.1111/vco.70024 (PMC12875758; doi:10.1111/vco.70024)

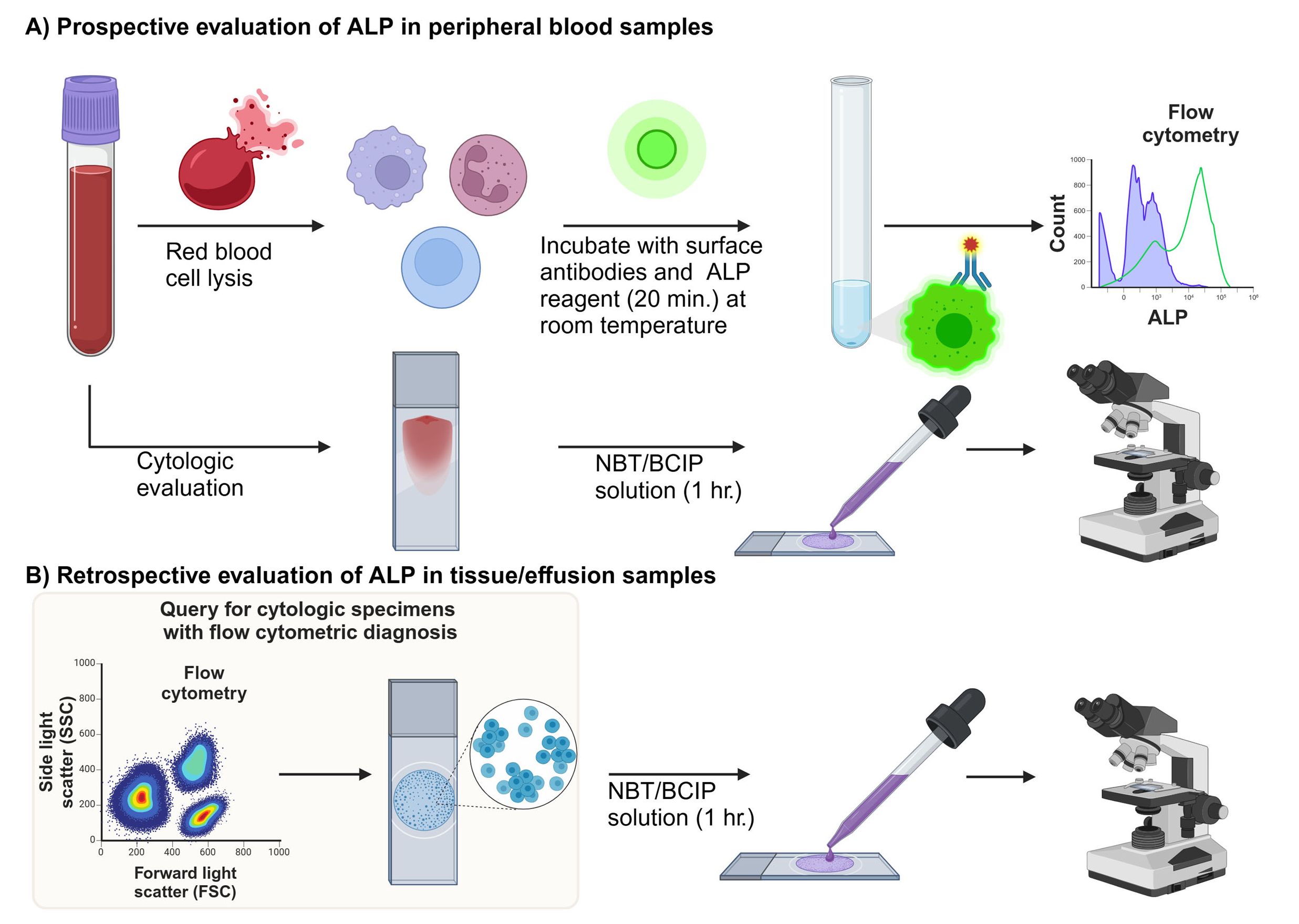

Supplement: Supplementary file 1 — Figure S1: Overview of methodology used in this study. (A) Prospective evaluation of ALP enzymatic activity in peripheral blood samples (normal, B cell CLL, CD34+ AL) from cases submitted to the Clinical Hematopathology Laboratory. Red blood cells were lysed, and remaining leukocytes were incubated with surface antibodies (Table S1) and ALP reagent for 20 min at room temperature. Cells were then analysed by flow cytometry to assess ALP expression, and cytologic evaluation was performed following staining with NBT/BCIP substrate solution for 1 h. (B) Retrospective evaluation of ALP enzymatic activity in tissue and effusion samples. Cytologic specimens with available flow cytometric diagnosis were identified, and ALP activity was evaluated using the NBT/BCIP staining method for 1 h, followed by microscopic analysis. Figure created in Biorender.com. [file VCO-24-41-s001.tiff]

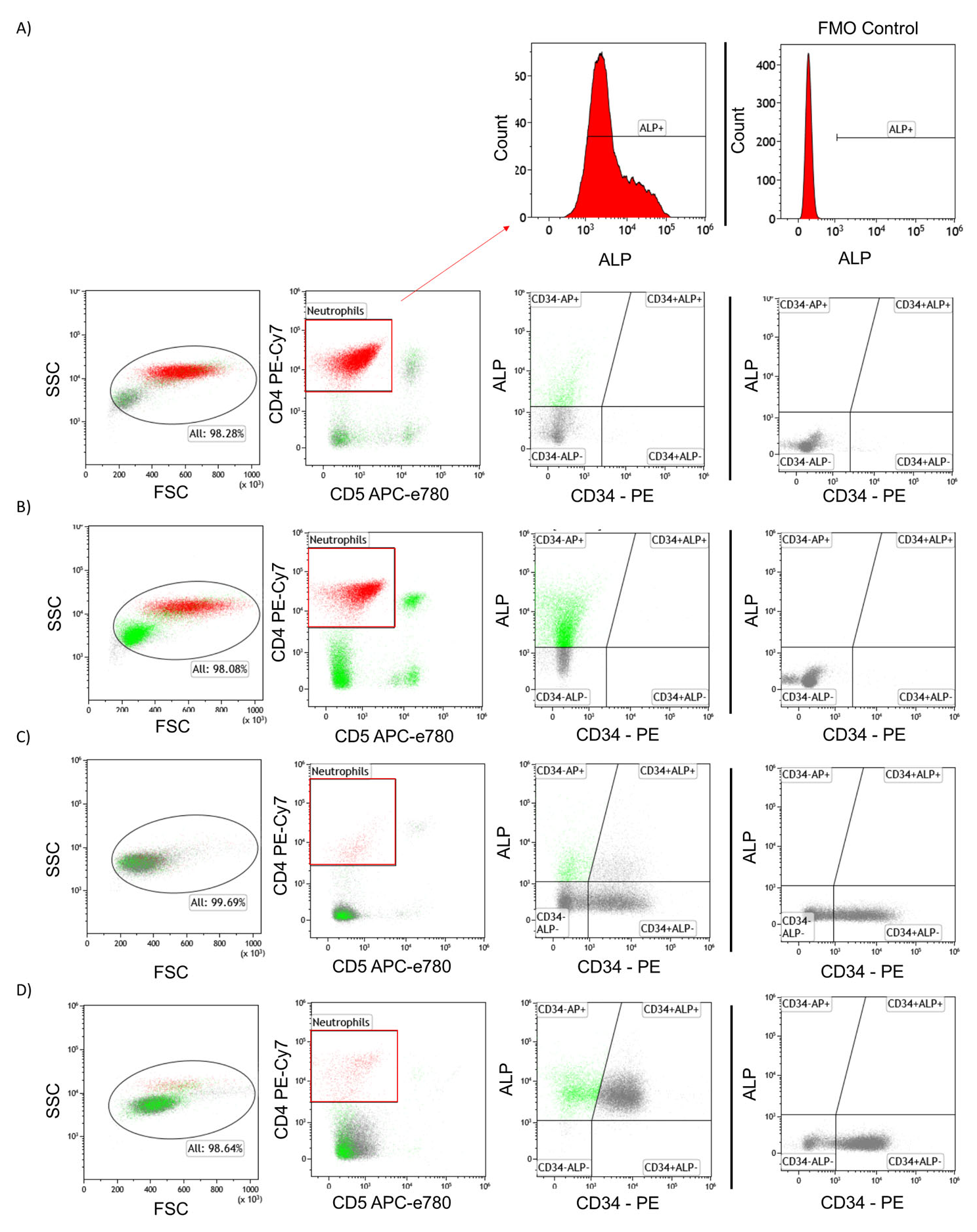

Supplement: Supplementary file 2 — Figure S2: Representative flow cytometric plots showing ALP expression in a normal control sample (A), a B cell CLL case (B), an AUL case (C) and an AML case (D). Each panel illustrates the gating strategy, including the exclusion of identifiable neutrophils (outlined in red) and the use of fluorescence‐minus‐one (FMO) controls (rightmost plots). (A) Topmost flow cytometry plots demonstrate ALP activity in neutrophils, which were excluded from downstream analysis. Despite the exclusion of neutrophils, ALP activity was detected in other peripheral blood leukocytes and across multiple sample types. Notably, CD34− ALP+ cells (highlighted in green) were observed, suggesting a broader distribution of ALP expression beyond neutrophils. [file VCO-24-41-s005.tiff]

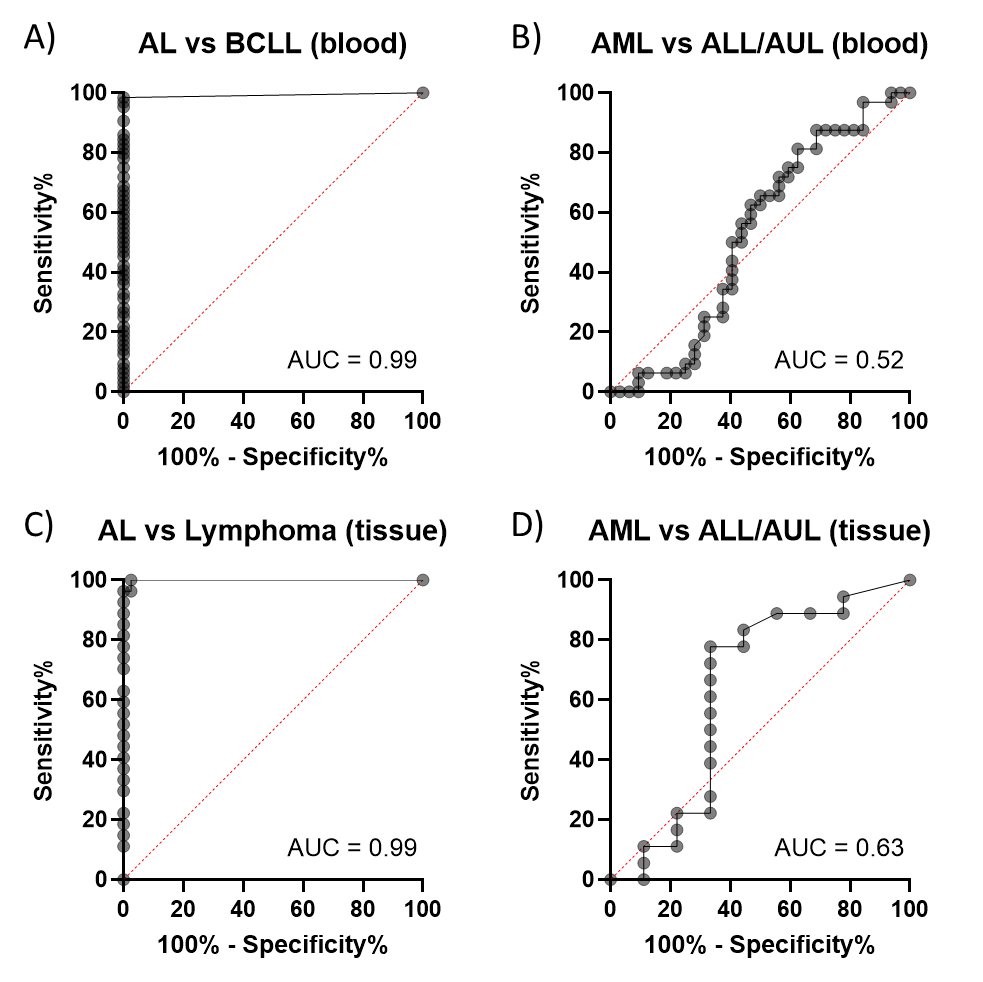

Supplement: Supplementary file 3 — Figure S3: Receiver operating characteristic (ROC) curves of ALP% determined by cytochemical staining for distinguishing acute leukaemia (AL) and acute myeloid leukaemia (AML) from other hematologic malignancies in blood and tissue samples. (A) Comparison of AL vs. B cell CLL (BCLL) in blood cases. (B) Comparison of AML vs. ALL/AUL in blood cases. (C) Comparison of AL vs. lymphoma using tissue samples. (D) Comparison of AML vs. ALL/AUL using tissue samples. Area under the curve (AUC) values are shown in each panel. [file VCO-24-41-s003.tif]

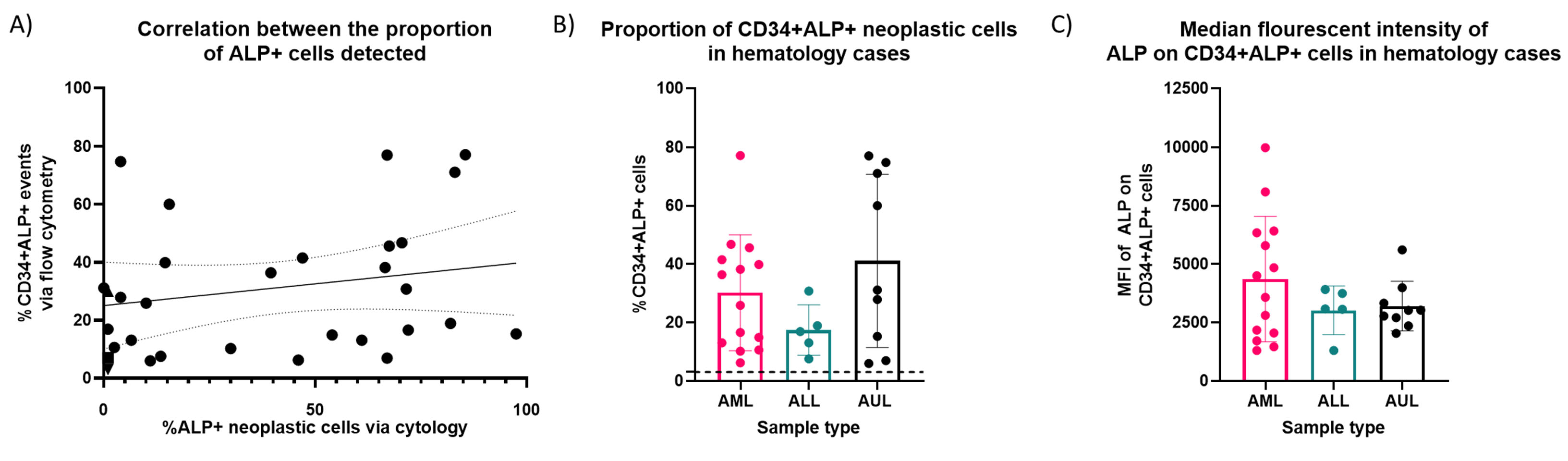

Supplement: Supplementary file 4 — Figure S4: Flow cytometric analysis of ALP expression in CD34+ AL cases. (A) Correlation between the percentage of ALP+ neoplastic cells identified by cytochemical staining and the percentage of CD34 + ALP+ cells detected by flow cytometry (Spearman's rho = 0.25). (B) Box plot comparing the proportion of CD34 + ALP+ cells between haematology cases. No statistically significant difference in the proportion of CD34 + ALP+ cells was observed between CD34+ AL subtypes (ANOVA p > 0.05). (C) Box plot highlighting the MFI of ALP on CD34 + ALP+ cells. No statistical difference was observed between CD34+ AL subtypes (ANOVA p > 0.05). [file VCO-24-41-s004.tiff]
